# Scions impact biomass allocation and root enzymatic activity of rootstocks in grafted melon and watermelon plants

**DOI:** 10.3389/fpls.2022.949086

**Published:** 2022-09-29

**Authors:** Glenda Sallaku, Boris Rewald, Hans Sandén, Astrit Balliu

**Affiliations:** ^1^ Faculty of Agriculture and Environment, Agricultural University of Tirana, Tirana, Albania; ^2^ Department of Forest and Soil Sciences, University of Natural Resources and Life Sciences, Vienna (BOKU), Vienna, Austria

**Keywords:** Cucurbitaceae, extracellular enzymes, grafting, rootstock-scion relationships, root traits

## Abstract

Vegetable grafting is increasingly recognized as an effective and sustainable plant production alternative. Grafted plants usually show increased uptake of water and minerals compared with self-rooted plants, mostly thought a consequence of the vigorous rootstocks selected. However, while studies frequently addressed the effects of rootstocks on the performance of scions, knowledge on the influences of scions on biomass allocation, morphology, and metabolic activity of roots is rare. In particular, the plasticity of root traits affecting resource acquisition and its efficiency remains poorly understood. Two different rootstock species, *Cucurbita maxima* × *Cucurbita moschata* and *Lagenaria siceraria*, were grafted in combination with melon (*Cucumis melo*) and watermelon (*Citrullus lanatus*). Self-grafted rootstocks were used as control. Plant biomass and root traits were determined after destructive harvesting 30 and/or 60 days after grafting. Traits included biomass allocation, leaf and root morphology, potential activities of four extracellular enzymes on root tips and basal root segments, and root respiration. Successfully grafted scions increase the ratio of root to whole plant dry matter (RMF), and increased ratios of root length to whole plant dry matter (RLR) and to plant leaf area (RL : LA). In contrast, morphological root traits such as diameter, tissue density, and specific root length remain surprisingly stable, and thus scion-induced changes of those traits may only play a minor role for the beneficial effects of grafting in Cucurbitaceae. Incompatibility in melon/*L. siceraria* grafts, however, was likely responsible for the reduced root growth in combination with clear changes in root morphological traits. Reduced root respiration rates seem to be the effects of a non-compatible rootstock–scion combination rather than an active, C-efficiency increasing acclimation. In contrast, heterografts with melon and watermelon frequently resulted in root-stock-specific, often enhanced potential enzymatic activities of acid phosphatase, *β*-glucosidase, leucine-amino-peptidase, and N-acetyl-glucosaminidase both at root tips and basal parts of lateral roots—presenting a potential and complementary mechanism of grafted plants to enhance nutrient foraging. The studied melon and watermelon scions may thus increase the nutrient foraging capacity of grafted plants by fostering the relative allocation of C to the root system, and enhancing the extracellular enzymatic activities governed by roots or their rhizobiome.

## Introduction

Vegetable crops are fast-growing, highly resource-demanding crops. Although the lack of nutrient availability is frequently a main cause of restricted plant growth (De Oliveira et al., 2018), the limited availability of arable land frequently requires their cultivation under unfavorable conditions (Sallaku et al., 2019). These include the limited availability of specific nutrients, with nitrogen (N) and phosphorus (P) as the most common limiting elements (Vitousek et al., 2010).

Plants have evolved a number of adaptive root traits that allow them to access different pools of resources (Wen et al., 2022). Mitigation strategies of plants in nutrient deficient soils range from changes in root morphology and architecture, symbiotic associations, metabolic adaptions to modified exudation of acid phosphatases, and organic anions (carboxylates) (Wang and Lambers, 2020; Freschet et al., 2021b); all entail carbon (C) costs (Li et al., 2011). For example, root exudation and respiration can consume 21% and 30% of total photosynthetic C allocated belowground, respectively (Sun et al., 2021). Hence, to maximize uptake at limited C availability, trade-offs between different acquisition strategies exists. For example, plants can optimize resource uptake by investing C either in thinner or less dense roots that efficiently explore the soil (Bergmann et al., 2020), or in mycorrhizal fungal partners (Bergmann et al., 2020), or adapt root metabolic activity, including more efficient nutrient recycling, increased uptake, or alterations in exudate composition and rate (Pant et al., 2008; Liu et al., 2011; Ling et al., 2013). Root exudation interacts with soil microbes and influences rhizosphere processes directly or indirectly (Williams et al., 2022). The extracellular enzymes, usually secreted by roots, their mycorrhizal symbionts, or associated microbes, and amplified when nutrient availability is low (Machado and Furlani, 2004; Meier et al., 2020), are surface-located, or surface-bound (Courty et al., 2005) and catalyze biochemical reactions, including nutrient cycles (Pritsch and Garbaye, 2011). Plant-exuded acid phosphatases (AP) or phosphomonoesterases are a group of extracellular enzymes involved in releasing phosphate groups from organic phosphates (Li et al., 2011; Lambers et al., 2013; Faucon et al., 2017). Symbionts and associated microbes of roots also exude a number of *β*-glucosidases (BG), which cleave the disaccharide cellobiose into two molecules of glucose (Sanaullah et al., 2016; Zhang et al., 2017). In addition, *β*-glucosidases influence plant’s chemical defense responses, activation of plant growth regulators, and lignification or cell-wall catabolism (Koudounas et al., 2015). L-Leucine aminopeptidases (LAP) are involved in nitrogen remobilization through hydrolysis of amino acid residues and protein degradation (Matsui et al., 2006; Mori et al., 2018), whereas *β*-1,4-N-acetyl-glucosaminidases (NAG) are involved in hydrolysis of chitin and finally release acetyl glucosamine (Mori et al., 2018). However, acquisition strategies and thus acclimation measures to mitigate low nutrient availabilities are often element specific (Freschet et al., 2021b).

In addition to genotype-intrinsic acclimation potentials, specific agro-technologies are applied to maximize growth and yield under given environments. Among them, grafting is increasingly (re-)considered an effective measure to cope with environmental constrains (Mauro et al., 2022). Grafting is an ancient agricultural practice that joins the root system (rootstock) of one plant to the shoot (scion) of another (Warschefsky et al., 2016). Although the main purpose of grafted seedlings was to increase the yield and quality of fruits by combining a disease-resistant rootstock with a genetically superior scion (Lee, 2007), improved tolerances to environmental stresses such as low soil temperatures, flooding, salinity and high heavy metal concentrations have been reported (Rivero et al., 2003; Edelstein et al., 2012; Fernández-Paz et al., 2021). As different grafting methods were developed (Balliu and Sallaku, 2017), grafting is now used at an unprecedented scale as an effective and sustainable plant production alternative (Rouphael et al., 2018; Reyes-Herrera et al., 2020). Grafted plants usually show increased uptake of water and minerals compared with self-rooted plants (Yuan et al., 2016). This was mostly attributed to the vigorous root system of commercial rootstocks (Kawaguchi et al., 2008; Martínez-Ballesta et al., 2010), e.g., increasing both the absorption and translocation of nutrients (Ruiz et al., 1997). Increased concentrations of specific nutrients in grafted vs. non-grafted plants were, e.g., reported for leaves and stems of grafted cucumber (Rouphael et al., 2008), melon (Ruiz et al., 1997), and watermelon (Colla et al., 2010a).

However, while many previous studies addressed the effects of different rootstocks on vegetative growth (Babaj et al., 2014a; Babaj et al., 2014b), stress tolerance (Savvas et al., 2010; Rouphael et al., 2018), yield (Balliu et al., 2008; Yuan et al., 2016), and fruit quality (Kyriacou et al., 2017) of scions, little is still known about possible influences of scions on the architecture and metabolic activity of the rootstocks (Neumann et al., 2014; Gautier et al., 2021). Furthermore, while a few published reports, to the best of our knowledge, address the effects of a scion cultivar on the rooting pattern of the rootstock (Franco et al., 1995; Harrison et al., 2016; Thomaj et al., 2019), potential eco-physiological alterations affecting the nutrient acquisition capacity and efficiency of rootstocks remain poorly understood (Shu et al., 2017). As it becomes more and more clear that scions modify rootstock responses to the environment in a genotype-specific manner (Gautier et al., 2020), the importance of studying grafted plants during rootstock selection becomes evident. Hence, the aim of this research is to assess the effect of grafting on root architectural, morphological, and metabolic (respiration, and potential enzyme activities) traits of two commercially important cucurbit rootstocks *Cucurbita maxima* × *Cucurbita moschata* and *Lagenaria siceraria—*providing novel insights into the plasticity of root traits as affected by scions (i.e., self-grafting, melon, and watermelon). We hypothesize that heterografts cause significant changes in rootstock traits compared to self-grafted plants but that effects largely differ between scion genotypes, and modify root biomass and morphological and physiological traits to different extents.

## Materials and methods

### Plant material and growing conditions

Two different species—bottle gourd/calabash, i.e., *L. siceraria* (Molina) Standl., cv. “Emphasis F1” (Ls; Syngenta, Basel, Switzerland) and the interspecific hybrid *Cucurbita maxima* Duchesne × *Cucurbita moschata* Duchesne, cv. “Benhur F1” (CC; Grainez Voltz, Loire-Authion, France)—were used as rootstocks. Both species are of broad economic importance (Karaağaç and Balkaya, 2013; Mashilo et al., 2022) and currently represents the most used species as rootstocks for grafted Cucurbitaceae plants. Each rootstock was grafted with two different cucurbit species, i.e., melon (M; *Cucumis melo* L.), cv. “Dikti RZ F1” (De Lier, The Netherlands), and watermelon [Wm; *Citrullus lanatus* (Thunb.) Matsum. & Nakai.], cv. “Lusitana RZ F1” (De Lier, The Netherlands). Self-grafted seedlings of each rootstock were simultaneously produced and used as controls; following the extensive review by Fullana-Pericàs et al. (2020), either non-grafted or self-grafted plants can be used as controls. Thus, six treatments, two self-grafted rootstocks (*C. maxima* × *C. moschata* and *L. siceraria*) and four heterografts of each scion species on each of the two rootstocks (*C. lanatus*/*C. maxima* × *C. moschata; C. melo*/*C. maxima* × *C. moschata; C. lanatus*/*L. siceraria; C. melo*/*L. siceraria*), were established (**Table 1**).

**Table 1:** Dry matter of roots (DM_root_), shoots (DM_shoot_), and the entire plant (DM_plant_) of graft combinations of the rootstocks *Cucurbita maxima* × *Cucurbita moschata* (CC) and *Lagenaria siceraria* (Ls), with melon (*Cucumis melo*; M), watermelon (*Citrullus lanatus*; Wm), or self-grafted at day 60 after grafting (DAG 60).

Graft combination (Scion/Rootstock)	Abbreviation	Dry matter (g plant^−1^)			Biomass allocation	
		DM_root_	DM_shoot_	DM_plant_	DM_root_ : DM_shoot_	RMF
*Cucurbita maxima × Cucurbita moschata*, self-grafted	CC	0.294 ± 0.013 c	3.567 ± 0.268 a	3.861 ± 0.279 a	0.23 ± 0.02 c	0.08 ± 0.00 c
*C. melo/C. maxima × C. moschata*	M/CC	0.297 ± 0.017 c	2.981 ± 0.137 b	3.279 ± 0.150 b	0.26 ± 0.01 b	0.09 ± 0.00 b
*C. lanatus/C. maxima × C. moschata*	Wm/CC	0.296 ± 0.012 c	2.962 ± 0.067 b	3.259 ± 0.073 b	0.29 ± 0.01 b	0.09 ± 0.00 b
*Lagenaria siceraria*, self-grafted	Ls	0.553 ± 0.035 a	2.956 ± 0.134 b	3.509 ± 0.159 a	0.32 ± 0.01 a	0.16 ± 0.01 a
*C. melo/L. siceraria*	M/Ls	0.018 ± 0.007 d	1.589 ± 0.139 d	1.608 ± 0.140 d	0.07 ± 0.01 d	0.03 ± 0.00 d
*C. lanatus/L. siceraria*	Wm/Ls	0.393 ± 0.019 b	2.295 ± 0.097 c	2.689 ± 0.116 c	0.35 ± 0.01 a	0.15 ± 0.00 a
Scion (Sc)		**<0.001**	**<0.001**	**<0.001**	**<0.001**	**<0.001**
Rootstock (Rst)		0.114	**<0.001**	**<0.001**	0.109	**<0.001**
Sc × Rst interactions		**<0.001**	**0.024**	**<0.001**	**<0.001**	**<0.001**

Biomass allocation at DAG 60 is indicated by the DM_root_ : DM_shoot_ ratio and the root mass fraction (RMF). Different letters indicate significant differences within parameters (Tukey test, p < 0.05; mean ± SE); significant p-values of a two-way ANOVA are indicated in bold. See **Supplementary Table S1** for data at DAG 30. Abbreviations of graft combinations used throughout the text are given.

Graded seeds of scions and rootstocks were sown in foam trays filled with a mixture of peat and vermiculite (75%, 25%; vol:vol; 100 cm^3^ per plant) in a plastic greenhouse. Fourteen days after sowing, grafting was performed on equally sized plants by the “cutting grafted” method (Shibuya et al., 2007; Babaj et al., 2014a). In this technique, grafted seedlings are obtained by grafting scions on rootstock cuttings harvested from seedlings, which are then planted in growing medium for rooting (Shibuya et al., 2007); see Balliu and Sallaku (2017) for a representative image of recent and rooted grafts obtained by the method. Immediately after grafting, planting trays were placed in a growth chamber (KBW 400, Keison Products, Essex, England), maintained at an air temperature of 26°C, and a relative humidity (RH) of 100% at three successive days; RH was then gradually decreased to 90%. PPFD was 100 µmol m^−2^ s^−1^ with a photoperiod of 12 h (white fluorescent lamps). After 10 days (DAG 10), the plants were transferred to a plastic greenhouse.

Thirty days after grafting (DAG 30), 20 healthy, homogeneously sized seedlings of each experimental treatment were individually transplanted into 2-L plastic pots (20 × 12 × 8 cm) filled with a homogenized, nutrient-poor mixture of loamy Cambisol soil (from Albania) and 0.8–2 mm quartz sand (vol:vol, 1:3) with a pH(CaCl_2_) 7.5, C 0.28%, and N 0.022%; quartz sand was added to facilitate the retrieval of undamaged roots. Equal quantities of a 20% Hoagland nutrient solution (Hoagland and Arnon, 1950) were supplied to each plant at three time points (approximately DAG 15, 30, and 45); tap water was applied regularly to maintain water availability close to field capacity.

### Plant sampling, growth analyses, and biomass allocation

Thirty (DAG 30) and sixty (DAG 60) days after grafting, 10 randomly selected plants per treatment were harvested and used for biomass assessment and growth analyses. For that purpose, immediately after harvesting, the root system was gently washed free of adhering soil particles using a soft water jet, and subsequently plants were separated into roots and shoots. Leaf and root morphological assessment and growth analyses were performed as described below. At DAG 60, the same plants (10 plants per treatment) were in addition used for the enzyme assay. At the same time, five other randomly selected plants for each treatment were harvested and used for root respiration assessment (see below).

The relative growth rates (RGRs) of roots, shoots, and the entire plant were calculated for the period DAG 30 to DAG 60. For that purpose, following the morphological and eco-physiological analyses, roots and shoots of all plants at each respective harvesting date were dried (65°C, 48 h). Dry matter (DM) was determined per plant (± 0.001 g). Based on ΔDM between DAG 30 and DAG 60, the relative growth rates of the root system (RGR_root_) and the whole plant (RGR_plant_) were calculated (Hoffmann and Poorter, 2002; Hunt, 2003). The ratio of root dry matter to shoot dry matter (DM_root_ : DM_shoot_; Freschet et al., 2015a) and the root mass fraction (RMF) expressed as root dry mass divided by whole plant dry mass (g g^−1^; Freschet et al., 2015b) were calculated.

### Leaf and root morphological analyses

The roots of 10 plants per treatment (DAG 60) were scanned (10000XL with transparency unit; Epson, Japan; gray scale, 300 dpi) and the acquired root images were analyzed with the software WinRhizo 2012b Pro (Regent Instruments Inc., Quebec, Canada). The following parameters were determined individually for each plant: root length (RL), root surface area (RSA), root volume (RV), and average diameter (AvgD). After scanning, the roots were dried (60°C, 48 h) and dry matter of roots (DM_root_) per plant was individually weighted (± 0.001 g). Based on these, the specific root length (SRL)—root length divided by root dry mass (m g^−1^), the root tissue density (RTD)—root dry mass divided by fresh root volume (g cm^−3^; Freschet et al., 2021a), and the root length ratio (RLR)—root length divided by whole plant dry mass (m g plant^−1^; Ryser, 1996) were calculated.

The third and fourth leaves of each plant were scanned (10000XL; Epson, Japan; gray scale, 300 dpi); the acquired leaf images were analyzed with the software WinFolia 2012 (Regent Instruments Inc., Quebec, Canada), and the leaf surface areas (LA) were individually recorded. The leaves were subsequently dried (60°C, 48 h) and weighed (± 0.001 g). The specific leaf area (SLA; cm^2^ g^−1^) was calculated for specific leaves (Freschet et al., 2015a); hereafter, total plant leaf area (LA; cm^2^) was estimated as a product of average SLA and plants’ total leaf DM. In addition, the RL-to-LA ratio (RL : LA) was calculated for each treatment.

### Potential enzyme activity

Ten plants of each experimental treatment (DAG 60) were used to assess potential enzyme activities (pEA_root_) on roots. Roots were gently washed free of adhering soil particles, and four root samples of about 1 cm length, two from the distal part of lateral roots (named as “tip”) and two others closer to the basal part of lateral roots (named “basal”), respectively, were sampled. Root sections were placed in reaction tubes filled with deionized water, and stored for 14–20 h at 4°C. pEA_root_ was performed on the dissected lateral root segments (i.e., tip or basal) according to the protocol of Courty et al. (2005) and its improvement in Pritsch et al. (2011)—using high-throughput photometric and fluorometric microplate assays. Substrates for enzymes as well as standard substrates were purchased from Sigma-Aldrich (MO, USA). The potential activities of four enzymes were measured, following the above protocols: *β*-glucosidase (BG, hydrolyses cellobiose into glucose), N-acetyl-glucosaminidase (NAG, breaks down chitin), acid phosphatase (AP, releases inorganic phosphate from organic matter), and leucine-amino-peptidase (LAP, breaks down polypeptides). 4-Methylumbelliferone (MUB) complexed substrates were used for BG (4-methulumbelliferyl *β*-D-glucopyranoside), NAG (4-methylumbelliferyl N-acetyl-β-D-glucosaminide), and AP (4-methylumbelliferyl phosphate), whereas 7-amino-4-methylcoumarin (AMC) was used for LAP (L-leucine-7-amido-4-methylcoumarin hydrochloride). Assay pH was 6.5 for LAP and pH 4.5 for other substrates. In brief, root segments were placed in a 96-well filter plate (30–40 μm mesh size; AcroPrep, Pall Life Sciences, Crailsheim, Germany) prefilled with 150 μl of rinsing buffer. To start the assay, the rinsing buffer was removed and discarded. Subsequently, 100 μl of the first substrate was added to the wells. The filter plate was placed on a horizontal shaker and dark-incubated; incubation times ranged between 15 and 70 min according to protocols. To stop the reaction, black-well plates were prefilled with 150 μl of stopping buffer; the substrate was vacuum-transferred into a black-well plate below. Standard curves containing either 50 μM MUB or 50 μM AMC (for LAP) solution were prepared on 96-well plates. The plate was closed with a cohesive plastic film and stored dark (20°C) until measurement. The filter plate was subsequently flushed (rinsing buffer) and the assay was repeated with other substrates. Fluorescence was measured using an EnSpire multimode-plate reader (PerkinElmer, USA) at 450 nm (at 365 nm excitation) at 20 and 100 flashes. pEA_root_ was calculated using regression curves following Pritsch et al. (2004). All root segments were finally scanned with a flatbed scanner, and analyzed for length, surface area, and volume. Subsequently, pEA at root tips and basal lateral root segments were expressed in pmol per unit of root surface area (pmol mm^−2^ min^−1^) and per unit of root volume (pmol mm^−3^ min^−1^).

### Root respiration

Five randomly selected plants were harvested (DAG 60); the root system was gently washed free of adhering soil particles and three root samples of about 1 g (fresh weight; plotted gently surface dry) per plant were taken for root respiration measurements. After sampling, the roots were kept for about 2 min in a water bath at 20°C for temperature acclimation and after that were sealed in plastic tubes (50 cm^3^) inside a climate cabinet at 20°C. The CO_2_ concentration inside the plastic tubes was measured by a portable CO_2_ gas analyzer (EGM-5, PP Systems, Amesbury, USA); CO_2_ concentration was recorded every 30 s at the interval 0–150 s. The received ppm CO_2_ values were plotted against time, and the slopes of respective linear regressions were used as an estimate of the root respiration rate (ppm CO_2_ s^−1^). Subsequently, the analyzed roots were scanned with a flatbed scanner and analyzed with the PC program WinRhizo 2012b Pro (as above). Finally, root respiration rate (RRR) was calculated per unit of root surface area (ppm CO_2_ s^−1^ cm^−2^).

### Statistical analyses

A factorial arrangement of six treatments in a randomized complete block design was used with 10 replicates each. Residuals of all variables were tested for equality of variances and normality using the tests after Brown–Forsythe and Shapiro–Wilk, respectively. When needed, specific traits were natural log-transformed to achieve normality. Differences in DM, RGR, and root traits were tested by one- or two-way ANOVA, using the PC program SigmaPlot v.13 (Systat Software Inc., San Jose, CA, USA). Each significant ANOVA result (*p* < 0.05) was followed by a *post-hoc* Tukey test at *p* < 0.05. Values given throughout the text are means ± standard error (SE). Pearson correlation coefficients were calculated to estimate the strength of relationships of resource acquisition root traits with AP, BG, LAP, and NAG pEA_root_, and the respective correlograms were performed *via* STHDA (2022). Furthermore, in order to produce a graphical evaluation of root trait relationship, a heatmap analysis was performed *via* the ClustVis (2022) program package.

## Results

### Dry matter partitioning, growth rate, and root morphology parameters

Strong reciprocal effects of scion and rootstock were found regarding dry matter of grafted plants (DM_plant_). At DAG 60, melon (M) and watermelon (Wm) grafted onto *L. siceraria* (Ls) and *C. maxima* × *C. moschata* (CC) featured significantly less DM_plant_ than the respective self-grafted rootstocks (**Table 1**). The reduction in DM_plant_ of plants grafted onto Ls was the result of significant reductions in both dry matter of shoots (DM_shoot_) and dry matter of roots (DM_root_). In contrast, DM_root_ remained stable in grafted CC, both by DAG 30 and by DAG 60. In CC, the entire reduction in DM_plant_ of heterografts was caused by a reduction in DM_shoot_ (**Table 1**; **Supplementary Table S1**). No difference existed between self-grafted Ls and CC regarding DM_plant_; however, melon and watermelon grafted onto Ls showed significantly less DM_plant_ than respective scions grafted onto CC rootstock (**Table 1**). Ls is characterized by a larger volume of thicker, less branched roots compared to CC (**Supplementary Figure S1**), which was reflected by a higher DM_root_ of self-grafted Ls seedlings. By DAG 30, DM_root_ of self-grafted Ls was 0.122 g plant^−1^, and by DAG 60, it reached 0.553 g plant^−1^, almost double the weight of roots in self-grafted CC (0.294 g plant^−1^) (**Table 1**; **Supplementary Table S1**). However, its heterografts with *C. melo* (M/Ls) or *C. lanatus* (Wm/Ls) held significantly less DM_root_, not only versus the self-grafted rootstock (Ls), but in case of M/Ls (0.018 g plant^−1^) also compared to M/CC. In contrast, Wm/Ls held a significantly greater DM_root_ compared to Wm/CC. No significant effects of scion on DM_root_ were identified in Wm/CC and M/CC graft combinations versus the self-grafted variant (CC; **Table 1**).

*L. siceraria* featured a significantly greater biomass allocation towards its root system than *C. maxima* × *C. moschata.* By DAG 30, DM_root_ of self-grafted Ls rootstock represented nearly 40% of dry matter weight of shoots, almost twice as high as in self-grafted CC (**Supplementary Table S1**). Although this proportion was gradually reduced, it remained high by DAG 60 (0.32)—significantly greater than in CC (**Table 1**). Similarly, RMFs of self-grafted Ls and Wm/Ls plants were significantly higher than that of CC plants (**Table 1**). Interestingly, DM_root_ : DM_shoot_ ratios of CC heterografts were significantly greater than those of self-grafted CC seedlings. No difference was found in case of grafted watermelon onto *L. siceraria*, either 30 or 60 days after grafting, but a strongly reduced root:shoot ratio was evident for M/Ls (**Table 1**, **Supplementary Table S1**).

**Figure 1:**
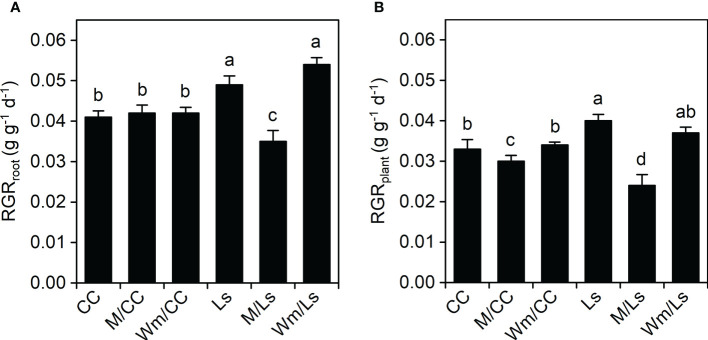
Relative growth rate of roots [RGR_root_, g g^−1^ day^−1^; **(A)**] and entire plants [RGR_plant_, g g^−1^ day^−1^; **(B)**] of self-grafted *Cucurbita maxima* × *Cucurbita moschata* (CC) and *Lagenaria siceraria* (Ls) and of respective heterograft’s with melon (M), and watermelon (Wm) between day 30 and 60 after grafting. Different letters indicate significant differences within RGR_root_ and RGRplant, respectively (Tukey test, *p* < 0.05; mean ± SE). See **Table 1** and **Supplementary Table S1** for biomass at DAG 60 and DAG 30, respectively.

*L. siceraria* grafts grew largely faster than *Cucumis maxima* × *Cucumis moschata*. In terms of RGR, self-grafted Ls and Wm/Ls possessed the highest RGR values at root and plant levels (**Figure 1**). RGRs of M/Ls plants were significantly less (0.0356 g g^−1^ plant^−1^ and 0.0243 g g^−1^ plant^−1^, respectively) compared to Ls and Wm/Ls. No differences were found among the three CC graft types regarding RGR_root_; however, RGR_plant_ of M/CC plants was significantly less compared to CC and Wm/CC (**Figure 1B**).

**Figure 2:**
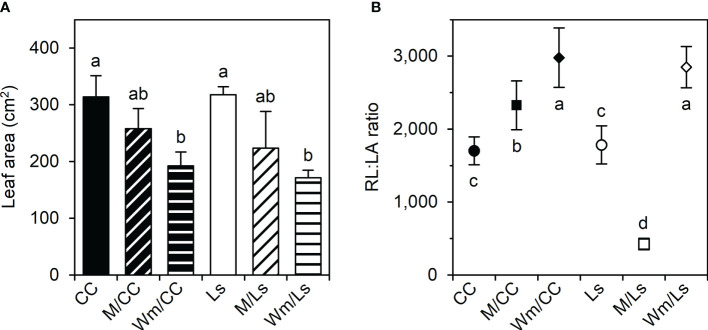
**(A)** Plant leaf area (LA, cm^2^) and **(B)** root length-to-leaf area ratio (RL : LA) of self-grafted *Cucurbita maxima* × *Cucurbita moschata* (CC), and *Lagenaria siceraria* (Ls) and of respective heterografts with melon (M) and watermelon (Wm) at day 60 after grafting (DAG 60). Different letters indicate significant differences within LA and RL : LA, respectively (Tukey test, *p* < 0.05; mean ± SE).

The leaf areas (LAs) of self-grafted rootstock’ species were greater than in heterografts, although this was only significant in grafts with Wm due to a high variability (**Figure 2A**). Thus, also no significant differences were found between the LA of grafted melon and watermelon within and between rootstocks. Very interestingly, however, the respective ratios of root length (RL) to plant’s leaf area (LA) of heterograft combinations were significantly higher than the respective ratios in self-grafted rootstocks; the exception being M/Ls, which had the significantly smallest ratio (**Figure 2B**).

No differences were found between self-grafted rootstocks (Ls and CC) regarding root length (RL) and root surface area (RSA). Self-grafted Ls had a significantly higher average root diameter (AvgD) and root volume (RV) compared with self-grafted CC. Ls plants were characterized by a much greater RTD and significantly greater root length ratio (RLR; **Table 2**). In contrast, CC plants had significantly greater SRL. Interestingly, heterografts only partially influenced the architectural and morphological root traits of the Ls rootstock, while there was largely no effect of heterografts on CC (the exception being RLR, which was significantly greater compared to CC; **Table 2**). Significant lower trait values of RL, RSA, AvgD, RV, and RLR were, however, found in M/Ls; SRL and RTD were significantly greater in M/Ls compared to self-grafted Ls plants (**Table 2**).

**Table 2:** Total root length (RL, cm), root surface area (RSA, cm^2^), root length ratio (RLR, m g^−1^), root average diameter (AvgD, mm), root volume (RV, cm^3^), root tissue density (RTD, mg^−1^ cm^−3^), and specific root length (SRL, cm g^−1^) of self-grafted *Cucurbita maxima* × *Cucurbita moschata* (CC), and *Lagenaria siceraria* (Ls) and of their heterografts with melon (M) and watermelon (Wm) at day 60 after grafting (DAG 60).

Graft combination (Scion/Rootstock)	RL (cm)	RSA (cm^2^)	RLR (m g^−1^)	AvgD (mm)	RV (cm^3^)	RTD (mg^−1^ cm^−3^)	SRL (cm g^−1^)
CC, self-grafted	4,790 ± 139 a	1,032 ± 29 a	12.97 ± 0.94 b	0.68 ± 0.014 b	17.78 ± 0.74 b	16.54 ± 0.49 b	166.42 ± 9.19 a
M/CC	4,994 ± 349 a	1,084 ± 64 a	15.27 ± 0.71 a	0.76 ± 0.064 ab	18.68 ± 1.04 b	15.70 ± 0.46 b	170.36 ± 8.34 a
Wm/CC	4,878 ± 201 a	1,066 ± 51 a	15.32 ± 0.54 a	0.69 ± 0.018 b	18.83 ± 1.25 b	16.41 ± 0.56 b	163.29 ± 6.96 a
Ls, self-grafted	4,986 ± 573 a	1,162 ± 96 a	14.26 ± 1.43 a	0.82 ± 0.052 a	21.85 ± 1.43 a	25.36 ± 0.46 b	90.08 ± 6.61 b
M/Ls	707 ± 42 b	78 ± 24 b	4.64 ± 0.49 c	0.48 ± 0.076 c	1.07 ± 0.34 c	64.42 ± 14.06 a	178.71 ± 8.29 a
Wm/Ls	4,435 ± 252 a	976 ± 38 a	16.00 ± 0.89 a	0.77 ± 0.063 ab	17.26 ± 0.71 b	23.86 ± 0.42 b	108.79 ± 6.44 b
Scion (Sc)	**<0.001**	**<0.001**	**<0.001**	**0.035**	**<0.001**	**0.001**	**<0.001**
Rootstock (Rst)	**<0.001**	**<0.001**	**<0.001**	0.619	**<0.001**	**<0.001**	**<0.001**
Sc × Rst interactions	**<0.001**	**<0.001**	**<0.001**	**<0.001**	**<0.001**	**<0.001**	**<0.001**

Different letters indicate significant differences within parameters (Tukey test, p < 0.05; mean ± SE); significant p-values of a two-way ANOVA are indicated in bold.

### Root respiration and potential enzymatic activity

**Figure 3:**
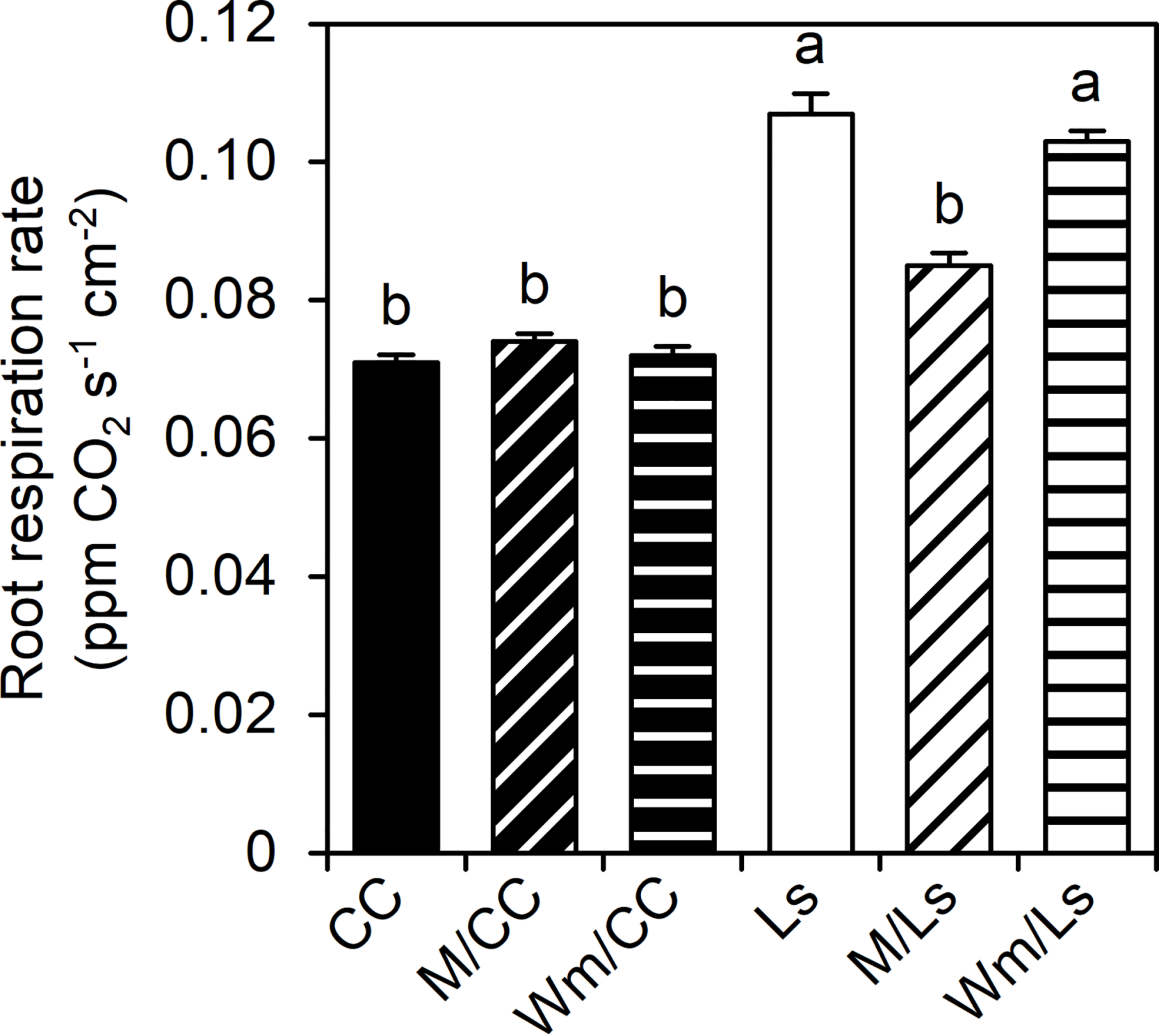
Root respiration rate per surface area (RRR, ppm CO_2_ s^−1^ cm^−2^) of self-grafted *Lagenaria siceraria* (Ls) and *Cucurbita maxima* × *Cucurbita moschata* (CC), and of respective heterografts with melon (M) and watermelon (Wm) at day 60 after grafting (DAG 60). Different letters indicate significant differences between RRR (Tukey test, *p* < 0.05; mean ± SE).

Ls and Wm/Ls plants possessed significantly greater RRRs per unit of root surface area (ppm CO_2_ s^–1^ cm^–2^) than CC graft types (**Figure 3**). While no effect of watermelon on Ls RRR was found, M grafted onto Ls significantly reduced RRR. No influence of scions (melon and watermelon) was found on the root respiration of CC rootstock (**Figure 3**).

**Figure 4:**
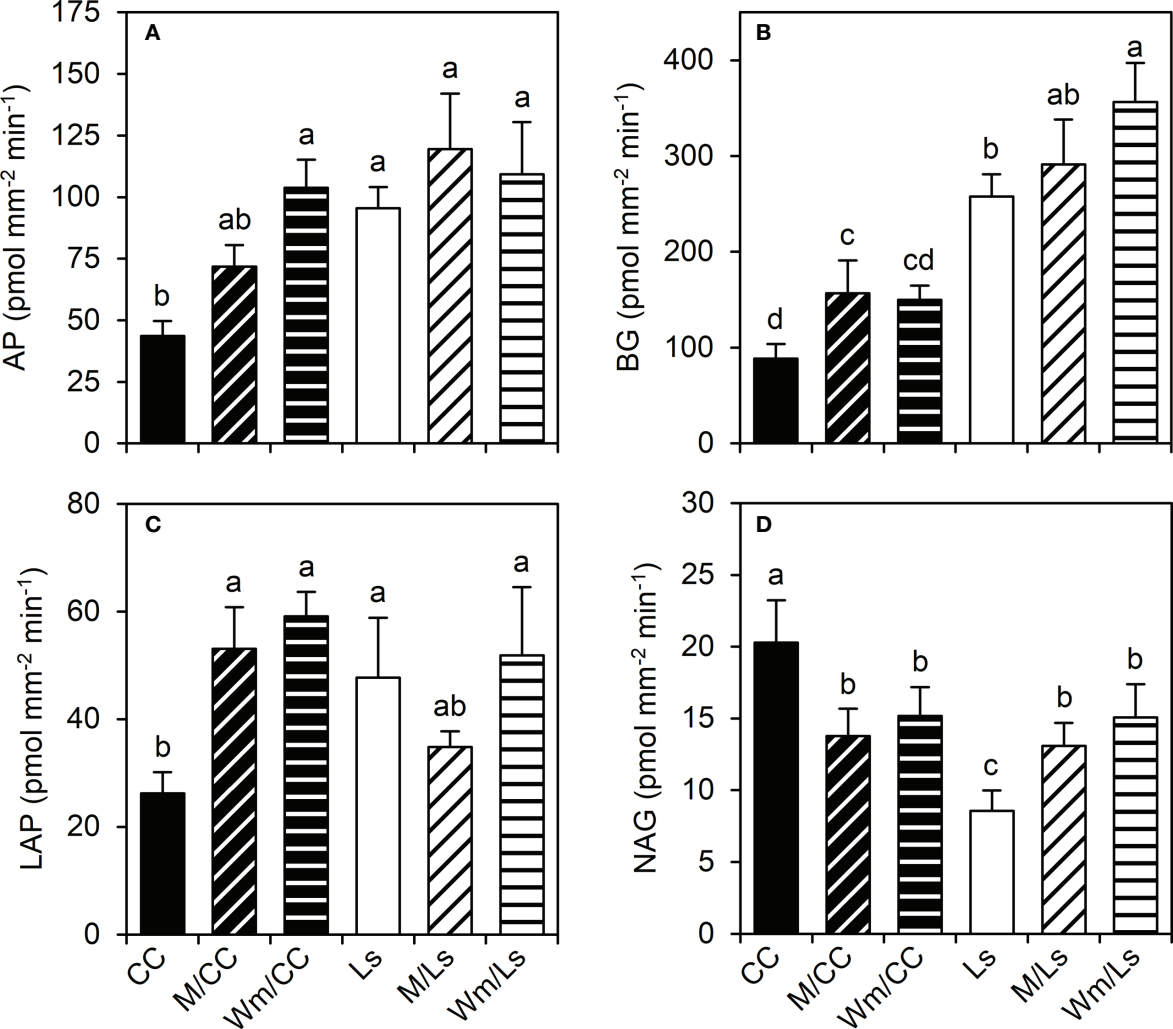
Potential enzyme activities (pEA_root_, pmol mm^−2^ min^−1^) of **(A)** acid phosphatase (AP), **(B)** β-glucosidase (BG), **(C)** leucine-amino-peptidase (LAP), and **(D)** N-acetyl-glucosaminidase (NAG) at root tips of different graft combinations of self-grafted *Cucurbita maxima* × *Cucurbita moschata* (CC) and *Lagenaria siceraria* (Ls), and of respective heterografts with melon (M) and watermelon (Wm) at day 60 after grafting (DAG 60). Different letters indicate significant differences per enzyme (Tukey test, *p* < 0.05; mean ± SE). See **Supplementary Table S2** for details including two-way ANOVA results, and **Supplementary Table S3** for pEA_root_ of basal root segments.

Significant statistical differences exist between *C. maxima × C. moschata* and *L. siceraria* regarding potential enzyme activities (pEA_root_) of both root tips and basal root segments of lateral roots. Ls holds significantly higher pEA_root_ of AP, BG, and LAP than CC (**Figures 4A–C**; **Supplementary Tables S2, S3**). A significantly lower NAG enzymatic activity of Ls compared to CC was found at root tips (**Figure 4D**) and basal root segments (**Supplementary Table S3**). Grafting melon or watermelon onto CC rootstock was followed by a significant increase in the pEA_root_ of AP, BG, and LAP of root tips (**Figure 4**). In contrast, grafting either M or Wm significantly reduced pEA_root_ of NAG in CC root segments of both types (**Figure 4**; **Supplementary Table S3**). Increased pEA_root_ were also noticed for two of the four enzymes (BG and NAG) when grafting M or Wm onto Ls rootstock; M/Ls tended (*p* < 0.1) to possess a slightly lower pEA_root_ of LAP, compared to self-grafted Ls plants (**Figure 4**). While it becomes apparent that heterografts affect pEA_root_ in both rootstocks, no significant differences between scion species (melon and watermelon) effects on pEA_root_ of AP, BG, LAP, and NAG were found. For root tips, however, the rootstock–scion interactions were statistically significant regarding LAP and NAG (**Supplementary Table S2**). Interestingly, the potential enzymatic activity was significantly dependent on the type of root segments, with root tips holding significantly greater pEA of all four extracellular enzymes than more basal root segments (**Table 3**). Although the differences between root tip and basal segments regarding potential activity of analyzed enzymes were only statistically significant when expressed per unit or root volume (pmol mm^−3^ min^−1^), the same trends were found when expressed per root surface (pmol mm^−2^ min^−1^, **Table 3**).

**Table 3:** Potential root enzyme activities (pEA_root_) of acid phosphatase (AP), β-glucosidase (BG), leucine-amino-peptidase (LAP), and N-acetyl-glucosaminidase (NAG) of lateral roots’ root tips and basal root segments in grafted cucurbit species.

	AP		BG		LAP		NAG	
	pEA_root_(SA), pmol mm^−2^ min^−1^	pEA_root_(RV), pmol mm^−3^ min^−1^	pEA_root_(SA), pmol mm^−2^ min^−1^	pEA_root_(RV), pmol mm^−3^ min^−1^	pEA_root_(SA), pmol mm^−2^ min^−1^	pEA_root_(RV), pmol mm^−3^ min^−1^	pEA_root_(SA), pmol mm^−2^ min^−1^	pEA_root_(RV), pmol mm^−3^ min^−1^
Root tip	89.2 ± 6.4 a	5,193 ± 374 a	217.9 ± 17.2 a	12,788 ± 1,050 a	45.2 ± 3.3 a	2,731 ± 192 a	14.3 ± 0.9 a	945 ± 89.5 a
Basal root segment	81.7 ± 5.6 a	3,830 ± 288 b	201.5 ± 19.5 a	9,630 ± 1,039 b	33.7 ± 2.1 b	1,447 ± 103 b	15.3 ± 1.1 a	636 ± 53.9 b
*p*-value	0.383	**0.005**	0.531	**0.035**	**0.004**	**<0.001**	0.512	**0.003**

pEA_root_ are expressed in either pmol per unit of root surface area [pEA_root_(SA), pmol mm^−2^ min^−1^] or per unit of root volume [pEA_root_(RV), pmol mm^−3^ min^−1^]. Different letters indicate significant differences within parameters significant p-values of the ANOVA are indicated in bold. (Holm-Sidak test, p < 0.05, n = 60; mean ± SE).

**Figure 5:**
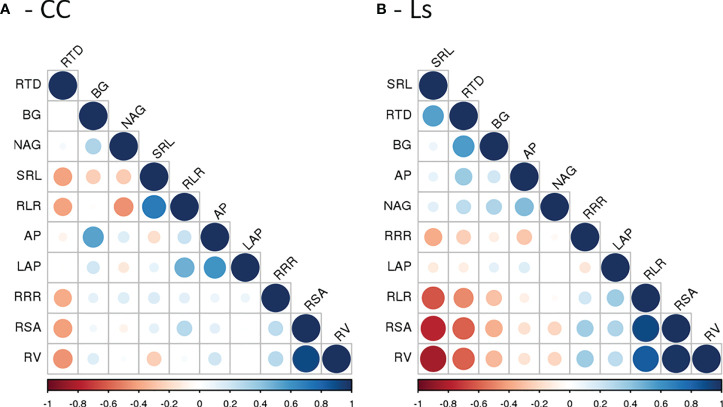
Pearson correlation matrix of morphological/architectural root traits (RV, RSA, RLR, RTD, and SRL), root respiration rate (RRR), and the potential root enzyme activities (AP, BG, LAP, and NAG) for **(A)** *Cucurbita maxima* × *Cucurbita moschata* and **(B)** *Lagenaria siceraria* self-grafted plants and respective heterografts with melon and watermelon. See text for details.

**Figure 6:**
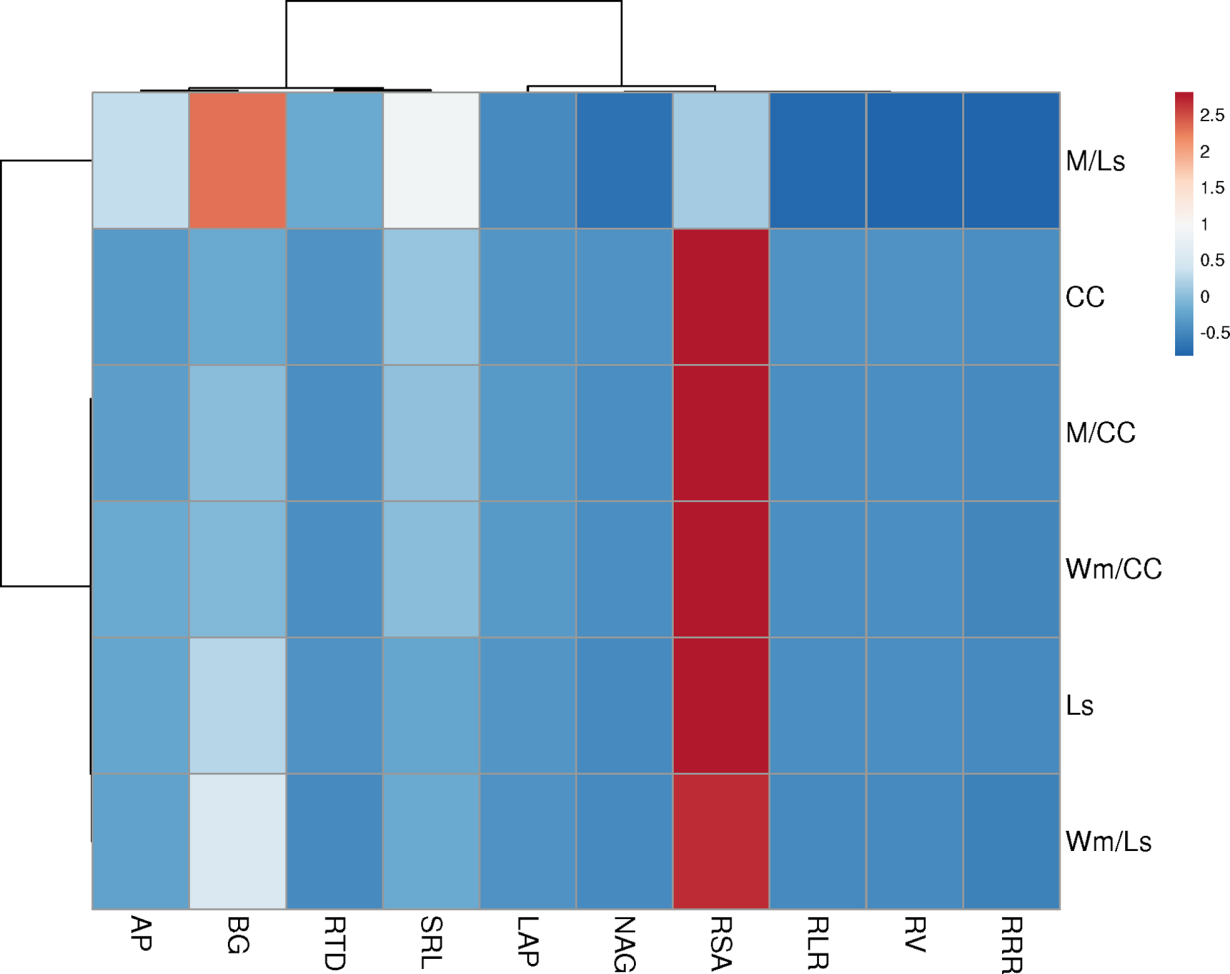
Clustered heatmap of root traits and enzyme exudation rate of AP, BG, LAP, and NAG of self-grafted *Cucurbita maxima* × *Cucurbita moschata* (CC), and *Lagenaria siceraria* (Ls) and of their heterografts with watermelon (Wm) and melon (M). Rows are centered; unit variance scaling is applied to rows. Both rows and columns are clustered using correlation distance and average linkage.

The relationships between resource-acquisitive root traits and pEA_root_ were represented by rather insignificant and mostly negative correlation coefficients (**Figure 5**). Moderate positive correlations between AP and BG pEA_root_ were only found with RTD in Ls rootstock plants (**Figure 5B**). A moderate positive correlation was also found between LAP pEA_root_ and RLR in CC rootstock plants (**Figure 5A**). In contrast, in the same plants, the moderate correlation between NAG pEA_root_ and RLR was negative. To our surprise, correlations between AP, BG, LAP, and NAG exudation rate with RRR were small and non-significant in both rootstocks (**Figure 5**). The heatmap analyses exposed a more comprehensive view of trait relationships (**Figure 6**). The respective clustered heatmap revealed two dendrograms; the one on the left site encompasses the groupings of the different grafts, whereas the one placed on the top clustered similar plant traits that affected the clustering of the different graft types. The left dendrogram shows a distinct separation of M/Ls’ root traits from the other grafts while CC graft types and Ls and Wm/Ls were sub-clustered as expected. Indeed, M/Ls shows lower values of RSA, RLR, RV, RRR, and NAG pEA_root_ and higher SRL, RTD, and AP and BG pEA_root_ values as described above. The main parameters that contribute in the separation of CC and Ls and M/Ls clusters were AP/BG pEA_root_ and RTD/SRL root traits. Specifically, the Ls cluster holds a higher BG pEA_root_ and smaller SRL values. Furthermore, CC is separated from M/CC and Wm/CC by differences in AP and BG pEA_root_ (**Figure 6**). The studied root traits were separated into two main subgroups. Most of them (RSA, RLR, RV, RRR, LAP, and NAG) are grouped together. However, within this subgroup, LAP is separated from the rest of the traits. The next main subgroup of parameters is composed of four traits: AP, BG, RTD, and SRL (**Figure 6**).

## Discussion

Commonly, grafting is considered a tool to boost the vegetative growth of scion cultivars, especially under adverse soil conditions (Colla et al., 2010a; Colla et al., 2010b; Schwarz et al., 2013; Böhm et al., 2017). Yet, in our experiment, the influence of scion on the rootstocks seems to be the opposite as compared with self-grafted *C. maxima* × *C. moschata* (CC) or *L. siceraria* (Ls), and DM_plant_ values of graft combinations of melon and watermelon with Ls and CC were significantly lower. In addition, differences regarding plant and root RGR were evidenced, especially in graft combinations of Ls with melon and watermelon. Together, this indicates that some degree of incompatibility exists between specific graft components. The incompatibility between rootstock and scion species is frequently explained by the presence of a necrotic layer at the graft junction and discontinuity in the vascular bundles at the graft union (Kawaguchi et al., 2008; Xu et al., 2015; Xiong et al., 2021), low level of antioxidant enzyme activities and high level of reactive oxygen species at the rootstock–scion interface (Aloni et al., 2008), the impairment of assimilate transport (Camalle et al., 2021; Xiong et al., 2021), and hormone synthesis and signaling (Camalle et al., 2021).

*C. maxima* × *C. moschata* rootstocks seem to show a higher degree of compatibility with melon and watermelon than Ls rootstocks. Whereas in heterograft combinations with Ls the reduction in DM_plant_ was a result of significant reductions in both DM_root_ and DM_shoot_, a stable DM_root_ was found in heterografts of CC. The latter is in contrast with previous studies, where large effects of scions on total rootstock biomass have been reported, e.g., for *Vitis* sp. (Tandonnet et al., 2010; Gautier et al., 2021). As the reduction in DM_plant_ of CC heterografts was caused by the reduction in DM_shoot_, we hypothesize that both melon and watermelon scions were able to supply sufficient photosynthetic assimilates to CC rootstock—maintaining DM_root_ at the level of self-grafted CC. Hence, although the DM_plant_ of CC heterografts with melon and watermelon was smaller than the DM_plant_ of self-grafted CC, rather than related with any incompatibility issue, this could be a consequence of a naturally smaller shoot dry weight of melon or watermelon plants compared with *C. maxima* × *C. moschata* shoots; however, self-grafted scions were not included in this study. Significant differences exist between melon and watermelon scions regarding compatibility with Ls, with the first one showing the lowest level of compatibility. Potentially, the melon scion was not able to supply carbohydrates to Ls rootstocks as effectively as self-grafted Ls. The reduced C availability in M/Ls rootstocks may also be evidenced by the significantly lower root respiration. Thus, while the entire plant growth was limited in M/Ls, this was particularly true for the root system, resulting in a dramatically reduced biomass allocation—which will have increasingly hampered water and nutrient supply to disproportionally growing shoots. Incompatibility issues between melon and bottle gourd (*L. siceraria*) were reported earlier (Xiong et al., 2021) and are confirmed by the findings of this experiment. Interestingly, both watermelon and melon scions have fostered root growth of CC versus the aboveground part, whereas no effect (watermelon) or a deleterious effect (melon) was found for the Ls root system. Thus, while self-grafted Ls and CC plants were characterized by a larger LA than the corresponding heterografts, the situation was the opposite regarding the respective ratios of root length to plant leaf area. Generally, a larger RL : LA ratio was recorded in heterografts, indicating an extended root length per unit leaf area and thus the ability to explore a (relatively) larger volume of soil. Since, RL : LA ratio reflects the plant’s structural expenditures for uptake (Körner and Renhardt, 1987), our data indicate the presence of a useful mechanism in successfully (i.e., compatible) grafted plants to increase the allocation of assimilates to rootstocks under limiting soil resources. In contrast, Gautier et al. (2021) reported only minor effects of specific *Vitis* scion/rootstock combinations on root-to-shoot fresh biomass ratios. However, previous studies on compatibility aspects among different Solanaceae (Kawaguchi et al., 2008) and also woody perennials such as apple trees (Thomaj et al., 2019) underlined that carbohydrate concentration and C partitioning between graft components are highly diverse and dependent on specific rootstock–scion combinations.

In general, no differences between Ls and CC regarding root length (RL) and root specific area (RSA) were observed. However, significant differences were found regarding root volume (RV), clearly a consequence of different average diameter of roots (AvgD). The significantly larger AvgD of Ls may indicate a greater potential for arbuscular mycorrhizal fungi colonization (Ma et al., 2018; Bergmann et al., 2020) than in CC roots, and potentially higher exudation rates (Williams et al., 2022) as supported by our data. Compared with self-grafted plants, AvgD remained unchanged in CC heterografts and in Ls and Wm/Ls types, but it was significantly reduced in M/Ls. Similarly, root diameters were not significantly altered by scion/rootstock combinations in *Vitis* genotypes (Gautier et al., 2021). Although not statistically significant, the reduced AvgD in M/Ls may be reflected by a slightly reduced LAP exudation rate of this variant (see also discussion below). While holding the shortest overall root length, as discussed above, M/Ls grafts possessed significantly greater SRL and RTD than self-grafted Ls. A higher RTD indicates a rather slowly grown root system (Bergmann et al., 2020)—in accordance with the measured RGR_root_. A greater tissue density is often the result of higher amounts of cell wall materials, or greater stele-to-cortex ratios, and often related to an increased root longevity (Ryser, 1996). As such, it seems that incompatible graft combinations adapt a slow growth strategy followed by an increased RTD to assure a longer root lifespan. Furthermore, since the production of finer roots is a strategy of plants to reduce root construction costs under low nutrient availability (Postma et al., 2014) and a high SRL can enhance nutrient acquisition by exploring a higher soil volume per unit of C investment in root length (Laliberté et al., 2015), by combining high RTD with increased SRL, the non-compatible M/Ls plants seem to have maximized carbon use efficiency. While not studied, we speculate that the reduced root respiration in M/Ls might, thus, beside the reduced availability of photosynthates discussed above, also be related to a lesser ratio of highly respiring cortex tissue. Certainly, plants can construct high SRL roots of any density, i.e., dense root tissue can be either thick or thin in diameter (Kramer-Walter et al., 2016), and this offers more possibilities for plants to better adapt to specific environmental conditions (Laughlin, 2014). In contrast to Ls, no differences were found between self-grafted CC and its heterograft combinations with melon and watermelon regarding SRL and RTD. Root tip density has not been accessed in our study, but modified branching frequencies will likely alter SRL and RTD. However, Gautier et al. (2021) also recently reported no significant effects of scions on root tip densities in *Vitis* sp. heterografts but strong effects of soil nutrient availability only. Further studies are required to determine the mechanisms resulting in anatomical and/or morphological changes in certain graft combinations under similar environmental conditions.

However, architectural or morphological adaptations might often not be sufficient to balance plants resource budgets: changes in metabolic activities, i.e., RRR (Amthor et al., 2019) and root and rhizosphere modifications to increase nutrient availability and uptake (Bilyera et al., 2022), are other alternatives. Significant differences exist between CC and Ls regarding RRR, with the latter one holding an about 20% higher RRR (beside M/Ls). While a reduction in root respiration helps to reduce C maintenance costs (Amthor et al., 2019; Lynch et al., 2021), it may also reduce phosphorus requirements (Fan et al., 2003) and thus help to maintain higher root growth rates (Walk et al., 2006; Postma et al., 2014). Therefore, a reduced RRR might be a useful plant adaptation of non-compatible graft combinations under low nutrient/phosphorus availability. However, in line with the findings that RRR is generally considered to be greater in fast-growing species compared with slow-growing ones (Rewald et al., 2014; Freschet et al., 2021a), RGR_root_ of the compatible grafts was matched by greater RRR in our study. Producing root exudates is part of the plant’s strategy to maximize acquisition in nutrient-deficient soils (Li et al., 2011; Lambers et al., 2013). Root exudates help plants face adverse conditions by inducing microbial responses (Sun et al., 2021), chelating toxic compounds from the soil, changing soil pH, or solubilizing nutrients (Vives-Peris et al., 2020). The majority of root metabolites are exuded at the root tip, where the lack of cell differentiation favors diffusion of metabolites to the soil (Sasse et al., 2018; Canarini et al., 2019)—potentially root tips and more basal root segments may also differ in their microbiome. We specifically found that root tips have a higher pEA_root_ of acid phosphatase (AP), *β*-glucosidase (BG), leucine-amino-peptidase (LAP), and N-acetyl-glucosaminidase (NAG) than the basal root segments. Similar, Bilyera et al. (2022) reported that AP activity was slightly greater at younger root tissue than at older ones. The further discussion is based on the enzymatic activity of root tips, which generally show the same pEA_root_ pattern between grafts as basal root segments. We found significant differences regarding root pEA between cucurbit rootstock species. While previous studies have largely addressed favorable nutrient uptake capacities of rootstocks (Lu et al., 2020), to the best of our knowledge, there is no other published evidence regarding vegetable rootstock’s root enzyme activity. However, it is already confirmed that differences exist among different herbaceous (Wen et al., 2019) and woody species (Sun et al., 2021). Very distinguishably, with the only exception of NAG, *L. siceraria* rootstocks are characterized by significantly higher pEA_root_ than *C. maxima* × *C. moschata*. The higher root pEA of AP and BG in Ls are in line with a previous report by Wen et al. (2019), concluding that species with thicker roots may release higher amounts of P-mobilizing exudates to the rhizosphere.

Very interestingly, we found that heterograft combinations of melon and watermelon, either onto CC or Ls, were characterized by significantly higher AP and BG pEA_root_ than the respective self-grafted rootstocks. The trend was the same with LAP and NAG, but here, significant scion–rootstock interactions resulted in an increase either in LAP (melon and watermelon grafted onto CC) or in NAG (melon or watermelon grafted onto Ls). Changes in root exudate profiles of bottle gourd (*L. siceraria*) grafted with watermelon were previously reported by Ling et al. (2013). Analyzing the content of chlorogenic and caffeic acid and their effects on plant susceptibility to *Fusarium oxysporum*, the authors found that the composition of the root exudates released by the grafted watermelon onto bottle gourd was different from both watermelon and bottle gourd plants. Song et al. (2016) reported a significantly higher diversity of root-secreted proteins in watermelon plants grafted onto bottle gourd compared with non-grafted watermelon and self-rooted bottle gourd. Similarly, by analyzing the influence of eggplants grafted onto a tomato rootstock, Liu et al. (2009) found differences regarding the composition of root exudates released between the non-grafted eggplants and tomato rootstock plants itself. Finally, Gautier et al. (2021) recently reported that heterografts of *Vitis* sp. can result in both unaltered and significantly increased AP activities compared to self-grafts. In sum, our study and previous ones suggest clearly that (some) scions co-regulate rootstock genotype-intrinsic exudation patterns. As established comprehensibly, the increased root exudation rate of organic acids (Colla et al., 2010a; Ling et al., 2013) and root enzymes (Pritsch and Garbaye, 2011; Lambers et al., 2013; Cenini et al., 2016) enhances plant nutrient uptake.

So far, there are a number of acclimation mechanisms reported in grafted vegetables to increase the nutrient acquisition vs. non-grafted seedlings, mostly related to the vigor of rootstocks in a specific graft combination. These include increased vigor of the root system and hence its capacity to explore wider and deeper soil volumes (Nawaz et al., 2016), alteration of macro- and micro-nutrient uptake rate (Nawaz et al., 2016; Ropokis et al., 2018; Yuan et al., 2016), enhanced specific nutrient absorption rate/efficiency (Sallaku et al., 2019), and enhanced exudation of organic acids (Colla et al., 2010a). In accordance, the results of this study clearly show that a compatible scion can enhance the relative nutrient foraging capacity by inducing changes in biomass allocation, i.e., increasing the ratio of root dry matter to whole plant dry matter (RMF), or improve the efficiency of carbon invested to root system by increasing the ratio of root length to whole plant dry matter (RLR), and/or increase the ratio of root length to plant leaf area (RL : LA), and the SRL. Similarly, scions have been reported to increase nutrient uptake efficiencies (Albacete et al., 2015; Gautier et al., 2021). Here, we report another potential mechanism, namely, a rootstock-specific increase in the potential activity of extracellular enzymes across the root system (root tips to basal root segments), to enhance the resource availability at the rhizoplane. Extracellular enzymes can be synthesized either by plant roots or their associated microbiome including arbuscular mycorrhiza (Cenini et al., 2016) and catalyze important biochemical reactions in the soil (Pritsch and Garbaye, 2011). Functions include hydrolysis and mobilization of P from a range of extracellular phosphomonoesters (acid phosphatases, AP) (Lambers et al., 2013), cellulose decomposition (*β*-1,4-glucosidase, BG) (Cenini et al., 2016), and N acquisition (L-leucine aminopeptidase, LAP; and *β*-1,4-N-acetyl-glucosaminidase, NAG) (Matsui et al., 2006; Cenini et al., 2016). As AP and BG clustered separately from the rest of analyzed traits, it seems that enhanced pEA_root_ of P mobilization (AP) and cellulose decomposition (BG) enzymes is a complementary strategy of both grafts to increase nutrient foraging in poor substrates. In contrast, pEA LAP and NAG were differentially affected in both rootstock by heterografts, potentially indicating different root or microbiome capacities to exude enzymes related to N acquisition.

Although negative correlations between root exudation rate and RTD are reported in trees (Sun et al., 2021) and in some grassland species (Williams et al., 2022), we found few and weak correlations of RTD with the potential enzymatic activity of AP, BG, LAP, and NAG. Also, our data do not support the conclusion by Sun et al. (2021) that exudation rate is positively correlated with root respiration. However, Sun et al. (2021) and Williams et al. (2022) calculated exudation rates as the total C exuded divided by root dry weight, whereas we measured the potential enzymatic activity of four specific root enzymes only. As exudation rates and the composition of root exudates are phylogenetically controlled (Williams et al., 2022) and highly species-specific (Wen et al., 2019), further studies on the role of plant- or microbiome-derived extracellular enzymes and other exudates for the nutrition of (grafted) Cucurbitaceae species are needed.

## Conclusion

Dry matter, root length, and root surface area of rootstocks in grafted cucurbitaceous plants are influenced by compatibility issues between scion and rootstock only. Incompatibility in M/Ls is responsible for reduced growth of the rootstock root system in combination with an increased SRL and a high RTD. Successfully grafted seedlings increased the RMF, and particular ratios of root length to whole plant dry matter (RLR) and root length to plant leaf area (RL : LA). In contrast, morphological parameters such as diameter, tissue density, and SRL remained surprisingly stable, and may thus only play a minor role for the beneficial effects of grafting. Similarly, a reduced RRR seems to be the effect of a non-compatible rootstock–scion combination rather than an adaptive acclimation. In contrast, grafting melon and watermelon onto *C. maxima* × *C. moschata* (CC) or *L. siceraria* (Ls) rootstocks resulted in root-stock-specific, often greater potential enzymatic activities of acid phosphatase (AP), *β*-glucosidase (BG), leucine-amino-peptidase (LAP), and N-acetyl-glucosaminidase (NAG) at both root tips and more basal parts of lateral roots. In sum, Cucurbitaceae scions may increase the nutrient foraging capacity of grafted plants by fostering the relative allocation of C to the root system, and/or enhancing its metabolic activity. As plants’ resource acquisition belowground can be modulated by a plethora of different root traits (Freschet et al., 2021b), interrelated to nutrient and C conservation strategies, future studies on grafting must include a larger set of not only root but also aboveground traits, and the associated rhizobiome—ranging from nutrient mobilization to uptake to internal recycling and regulation—enhancing our mechanistic understanding of this promising agro-technology.

## Data availability statement

The raw data supporting the conclusions of this article will be made available by the authors, without undue reservation.

## Author contributions

GS, BR, HS, and AB contributed to conception and design of the study. GS and AB performed the experimental setup and measurements. AB and HS organized the data and performed the statistical analysis. AB wrote the first draft of the manuscript. GS, HS, and BR wrote sections of the manuscript. All authors contributed to manuscript revision, read, and approved the submitted version.

## Funding

GS received a scholarship within the Jesh—Joint Excellence in Science and Humanities program (OEAW, Austria). BR and HS were partially funded by the University of Natural Resources and Life Sciences, Vienna (BOKU).

## Acknowledgments

We gratefully acknowledge the skillful contribution of Melanie Zillinger to pEA measurements. The authors thank three reviewers and the editor for helpful comments on an earlier version of the manuscript.

## Conflict of interest

The authors declare that the research was conducted in the absence of any commercial or financial relationships that could be construed as a potential conflict of interest.

## Publisher’s note

All claims expressed in this article are solely those of the authors and do not necessarily represent those of their affiliated organizations, or those of the publisher, the editors and the reviewers. Any product that may be evaluated in this article, or claim that may be made by its manufacturer, is not guaranteed or endorsed by the publisher.
