# Supplementary material for: Scions impact biomass allocation and root enzymatic activity of rootstocks in grafted melon and watermelon plants
Source: Front Plant Sci. 2022 Sep 29;13:949086. doi: 10.3389/fpls.2022.949086 (PMC9558002; doi:10.3389/fpls.2022.949086)
Supplement: Supplementary file 1 [file DataSheet_1.pdf]

Supplementary Material

1 Supplementary Tables

Supplementary Table S1. Dry matter of roots (DM_{root}), shoots (DM_{shoo}t), and the entire plant (DM_{plant}) for graft combinations of the rootstocks *Lagenaria siceraria* (Ls), and *Cucurbita maxima* × *Cucurbita moschata* (CC) with melon (*Cucumis melo*; M), watermelon (*Citrullus lanatus*; Wm), or self-grafted at day 30 after grafting (DAG 30). Biomass allocation is indicated by the DM_{root}:DM_{shoot} ratio. Different letters indicate significant differences within parameters (Tukey test, $p < 0.05$; mean±SE); significant p-values of a two-way ANOVA are indicated in bold.

Graft combination	Dry matter ($g\ plant^{-1}$) - DAG 30			
	**DM_{root}**	**DM_{shoot}**	**DM_{plant}**	**DM_{root} :DM_{shoot}**
CC, self-grafted	0.084±0.004 b	1.285±0.129 a	1.370±0.134 a	0.20±0.010 b
M/CC	0.081±0.006 b	1.226±0.102 a	1.308±0.107 a	0.31±0.024 a
Wm/CC	0.082±0.007 b	1.062±0.076 a	1.144±0.082 a	0.33±0.016 a
Ls, self-grafted	0.122±0.014 a	0.923±0.098 b	1.046±0.112 b	0.39±0.016 a
M/Ls	0.006±0.001 c	0.700±0.055 c	0.706±0.056 c	0.08±0.035 c
Wm/Ls	0.075±0.011 b	0.797±0.039 c	0.872±0.048 c	0.38±0.011 a
Scion (Sc)	**<0.001**	0.123	0.062	**<0.001**
Rootstock (Rst)	**0.045**	**<0.001**	**<0.001**	0.803
Sc × Rst interactions	**<0.001**	0.337	0.188	**<0.001**

Supplementary Table S2. Potential enzyme activities (pEA_{root}; pmol mm^{-2} min^{-1}) of acid phosphatase (AP), *β*-glucosidase (BG), leucine-amino-peptidase (LAP), and N-acetyl-glucosaminidase (NAG) of root tips of different graft combinations of self-grafted *Lagenaria siceraria* (Ls) and *Cucurbita maxima* × *Cucurbita moschata* (CC), and of their heterograft's with melon (M) and watermelon (Wm) 60 days after grafting (DAG 60). Different letters indicate significant differences within parameters (Tukey test, p <0.05; mean±SE); significant p-values of a two-way ANOVA are indicated in bold.

**Graft combination**	**pEA_{root} (pmol mm^{-2} min^{-1}) – root tips**			
	**AP**	**BG**	**LAP**	**NAG**
CC, self-grafted	43.67±13.80 b	88.57±31.10 d	26.18±7.52 b	20.29±2.11 a
M/CC	71.75±13.18 ab	156.69±29.65 c	52.33±7.17 a	13.76±2.01 b
Wm/CC	103.74±14.50 a	149.86±32.78 cd	60.75±7.92 a	15.19±2.22 b
Ls, self-grafted	95.56±13.80 a	257.60±31.10 b	47.73±7.52 a	8.55±2.11 c
M/Ls	119.55±13.18 a	291.18±32.78 ab	34.36±7.92 ab	13.08±2.36 b
Wm/Ls	109.31±14.5 a	356.30±29.65 a	50.43±7.92 a	15.09±2.01 b
Scion (Sc)	**0.034**	**0.042**	0.060	0.727
Rootstock (Rst)	**0.040**	**<0.001**	0.721	**0.021**
Sc × Rst interactions	0.212	0.520	**0.028**	**0.013**

Supplementary Table S3. Potential enzyme activities (pEA$_{root}$; pmol mm^{-2} min^{-1}) of acid phosphatase (AP), *β*-glucosidase (BG), leucine-amino-peptidase (LAP), and N-acetyl-glucosaminidase (NAG) of basal lateral root segments of different graft combinations (of self-grafted *Lagenaria siceraria* (Ls) and *Cucurbita maxima* × *Cucurbita moschata* (CC), and of their heterograft's with watermelon (Wm) and melon (M) at 60 day after grafting (DAG 60). Different letters indicate significant differences within parameters (Tukey test, p <0.05; mean±SE); significant p-values of a two-way ANOVA are indicated in bold.

**Graft combinations**	**pEA$_{root}$ (pmol mm^{-2} min^{-1}) – basal roots**			
	**AP**	**BG**	**LAP**	**NAG**
CC, self-grafted	59.76± 7.24 b	67.75±12.71 b	20.22±2.03 a	10.88±1.57 b
M/CC	57.86± 9.37 b	105.11± 9.67 b	32.03±6.63 a	10.10±0.65 b
Wm/CC	64.33±10.66 b	125.18±16.96 b	39.27±6.09 a	12.60±2.44 b
Ls, self-grafted	107.32±15.06 a	304.67±23.39 a	37.67±3.77 a	15.73±2.52 b
M/Ls	111.70±14.64 a	354.86±57.43 a	41.95±5.86 a	25.08±3.58 a
Wm/Ls	94.11±15.87 a	293.81±20.35 a	32.97±3.33 a	18.14±2.97 b
Scion (Sc)	0.898	0.408	0.206	0.234
Rootstock (Rst)	**<0.001**	**<0.001**	0.085	**<0.001**
Sc × Rst interactions	0.613	0.429	0.055	0.085

2 Supplementary Figures

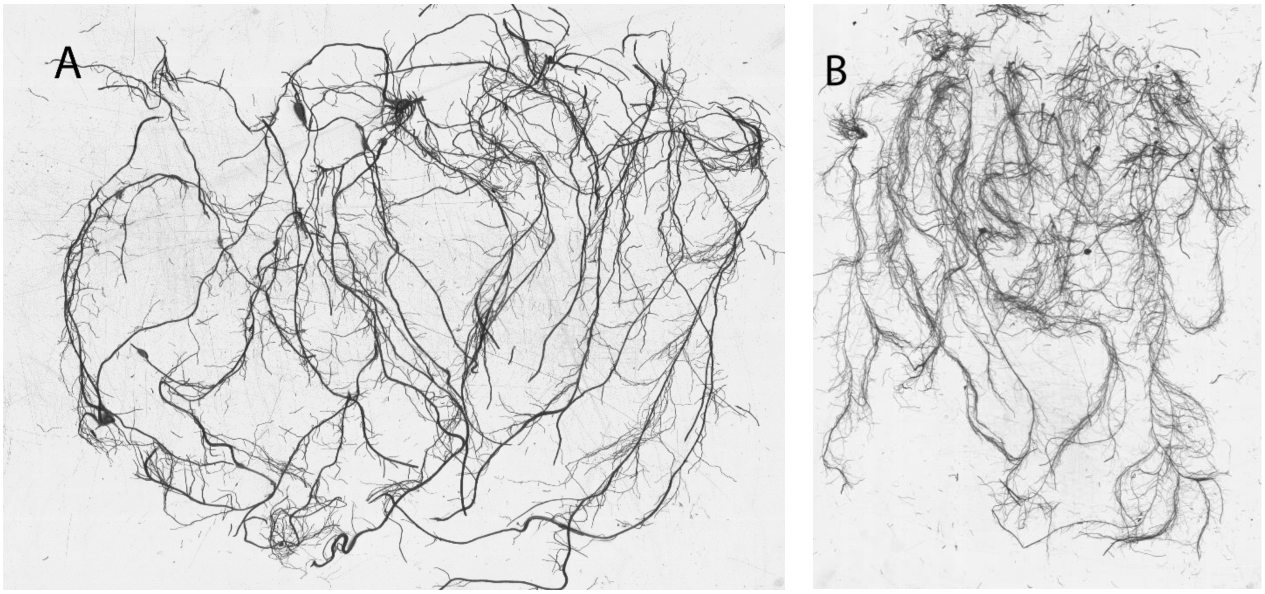

Supplementary Figure S1. Root systems of *Lagenaria siceraria* (A) and root system of *Cucurbita maxima* × *Cucurbita moschata* (B), both self-grafted
